# Field dipolarization in Saturn's magnetotail with planetward ion flows and energetic particle flow bursts: Evidence of quasi‐steady reconnection

**DOI:** 10.1002/2015JA020995

**Published:** 2015-05-19

**Authors:** C. M. Jackman, M. F. Thomsen, D. G. Mitchell, N. Sergis, C. S. Arridge, M. Felici, S. V. Badman, C. Paranicas, X. Jia, G. B. Hospodarksy, M. Andriopoulou, K. K. Khurana, A. W. Smith, M. K. Dougherty

**Affiliations:** ^1^ School of Physics and AstronomyUniversity of SouthamptonSouthamptonUK; ^2^ Planetary Science InstituteTucsonArizonaUSA; ^3^ The Johns Hopkins University Applied Physics LaboratoryLaurelMarylandUSA; ^4^ Academy of AthensAthensGreece; ^5^ Department of PhysicsLancaster UniversityBailriggUK; ^6^ Mullard Space Science LaboratoryUniversity College LondonSurreyUK; ^7^ The Centre for Planetary Sciences at UCL/BirkbeckLondonUK; ^8^ Atmospheric, Oceanic and Space SciencesUniversity of MichiganAnn ArborMichiganUSA; ^9^ Department of Physics and AstronomyUniversity of IowaIowa CityIowaUSA; ^10^ Space Research InstituteAustrian Academy of SciencesGrazAustria; ^11^ Institute of Geophysics and Planetary PhysicsUniversity of CaliforniaLos AngelesCaliforniaUSA; ^12^ Blackett LaboratoryImperial College LondonLondonUK

**Keywords:** Saturn, magnetotail, reconnection, beams

## Abstract

We present a case study of an event from 20 August (day 232) of 2006, when the Cassini spacecraft was sampling the region near 32 *R_S_* and 22 h LT in Saturn's magnetotail. Cassini observed a strong northward‐to‐southward turning of the magnetic field, which is interpreted as the signature of dipolarization of the field as seen by the spacecraft planetward of the reconnection X line. This event was accompanied by very rapid (up to ~1500 km s^−1^) thermal plasma flow toward the planet. At energies above 28 keV, energetic hydrogen and oxygen ion flow bursts were observed to stream planetward from a reconnection site downtail of the spacecraft. Meanwhile, a strong field‐aligned beam of energetic hydrogen was also observed to stream tailward, likely from an ionospheric source. Saturn kilometric radiation emissions were stimulated shortly after the observation of the dipolarization. We discuss the field, plasma, energetic particle, and radio observations in the context of the impact this reconnection event had on global magnetospheric dynamics.

## Key Points

- Rare observation of strong planetward flow following Saturn tail reconnection
- Ion flows at speeds of up to 1500 km/s observed, indicating energetic event
- Data imply quasi‐steady Vasyliunas‐type reconnection beyond 32 *R_S_*

## 1 Introduction

Reconnection in planetary magnetotails can result in significant reconfiguration of the magnetic field, acceleration of plasma flows, and energization of charged particles. Such changes can be measured in situ by orbiting spacecraft and remotely sensed through auroral imaging, mapping of energetic neutral atoms (ENAs), or measurement of radio emissions. Tailward of the X line, we may expect field and plasma to be broken off in the form of “plasmoids,” lumps of field and plasma that are ultimately free to escape downtail. Planetward of the X line, we may expect that the field will dipolarize, as closed field lines snap back toward the planet following reconnection, crossing the equatorial plane at shorter radial distances.

Much work has been published on the properties of dipolarizations at Earth, from analysis of the (often asymmetric) bipolar magnetic field signature of the dipolarization front [e.g., *Ohtani et al*., 2004; *Runov et al*., 2009] to the pressure, density, and temperature changes associated with inward moving plasma populations [e.g., *Sergeev et al*., 2009; *Runov et al*., 2011], and the bulk flow patterns that follow energetic reconnection events [e.g., *Lui et al*., 1977; *Schindler and Birn*, 1987; *Angelopoulos et al*., 1994; *Paterson et al*., 1998; *Raj et al*., 2002]. Intense beams are commonly observed in the outer plasma sheet/plasma sheet boundary layer (PSBL) during the substorm recovery phase, with the energy and strength of beams exhibiting latitude dependence, where beams observed closer to the lobes are faster and weaker than those near the equator [e.g. *Onsager et al*., 1990, 1991] (where speed variations are interpreted as a time‐of‐flight effect on recently reconnected field lines).

Recently, *Sundberg et al*. [2012] explored the characteristics of several dipolarizations in Mercury's tail using data from the MESSENGER spacecraft. They comment on many similarities between dipolarization fronts at Mercury and Earth, with hermean dipolarization field signatures displaying a sharp, rapid increase followed by a slower return to preonset values and with expected nonadiabatic heating of the plasma sheet (as the ion cyclotron period is on the order of the dipolarization time scale). Differences in spatial scales can arise due to the small size of Mercury's magnetosphere, while the short lifetime of events is attributed to the lack of steady field‐aligned current systems at Mercury, in the absence of an ionosphere in which to close currents.

At Jupiter, the situation is different again. In particular, the role of lobe reconnection in flux closure in the Jovian system is a subject of some debate [e.g., *McComas and Bagenal*, 2007; *Cowley et al*., 2008], while most recently, *Vogt et al*. [2014] estimated that the average reconnection event in Jupiter's tail closes 4–8 GWb of flux, ~1% of the typical ~720 GWb of flux contained in the Jovian tail [*Vogt et al*., 2011]. *Kasahara et al*. [2011, 2013] observed dipolarization fronts in the Jovian tail and interpreted decreases in plasma density, dropouts in energetic particle fluxes, and increases in reconnection outflow speed as evidence of transition from closed (plasma sheet) to open (lobe) field line reconnection at Jupiter. *Kronberg et al*. [2012], using data from the Galileo Energetic Particle Detector, observed field‐aligned beams of ions and electrons associated with reconnection jets.

Saturn represents a unique parameter space for dipolarizations: unlike the Earth or Mercury, rapid planetary rotation and internal plasma loading strongly influence magnetospheric dynamics, although perhaps not to the same extent as at Jupiter. Several studies have explored the effect of reconnection in Saturn's tail both in terms of local changes and more global influence. In particular, several authors have examined plasmoids and traveling compression regions (TCRs) tailward of the reconnection site [e.g., *Jackman et al*., 2007, 2008, 2014; *Hill et al*., 2008] using magnetometer and plasma data. Thus far, the study of the response of the region planetward of the X line has been somewhat limited [e.g., *Bunce et al*., 2005; *Thomsen et al*., 2013; *Mitchell et al*., 2014] and may represent an important “missing piece of the puzzle” for our understanding of the role of magnetic reconnection in Saturn's global magnetospheric dynamics.

The first published example of a dipolarization following reconnection at Saturn was from *Bunce et al*. [2005] who reported a reduction in total field strength, a change in field orientation indicative of a dipolarization, an intensification and low‐frequency extension of the Saturn kilometric radiation (SKR) emission, and a significant heating and energization of the local plasma. They interpreted these observations, made from a radial distance of ~16 *R_S_* (1 *R_S_* = 60268 km) and a local time of 3.6 h, as evidence of a solar wind compression‐induced reconnection event on the outbound pass of the Cassini Saturn Orbit Insertion maneuver. A further example of a dipolarization was presented by *Russell et al*. [2008], who examined the magnetic field data associated with the reconfiguration from a spacecraft position at 29.4 *R_S_* downtail and 01:36 LT. *Jackman et al*. [2013] presented another event and discussed the expected auroral counterpart of field dipolarizations, in the form of discrete spots formed by diversion of the cross‐tail current into the ionosphere via a scenario analogous to the terrestrial substorm current wedge [*McPherron et al*., 1973]. Most recently, *Thomsen et al*. [2013] surveyed Saturn's duskside magnetotail, finding occasional evidence of plasma flowing toward the planet, and presenting one particular case study of a dipolarization, including the first description of the corresponding plasma data at Saturn for such an event.

The question of what type of magnetospheric circulation leads to the observed signatures of reconnection is an open one at Saturn. The Vasyliunas cycle is internally driven and involves reconnection of closed field lines [*Vasyliunas*, 1983], while the Dungey cycle is driven by interaction with the solar wind and hence involves reconnection of open field lines [*Dungey*, 1961]. Attempts have been made to differentiate between the two types of reconnection in terms of field and plasma signatures [e.g., *Jackman et al*., 2011; *Masters et al*., 2011] and in terms of theoretical predictions for flow composition [e.g., *Cowley et al*., 2005; *Badman and Cowley*, 2007; *Thomsen et al*., 2013]. The plasma on closed field lines might be expected to include heavy, water group ions from the moon Enceladus, while the plasma on open field lines might be expected to consist primarily of protons and/or helium ions from the solar wind [e.g., *Young et al*., 2005]. Most recently, *Thomsen et al*. [2014] explored the statistical distribution of plasma flows in Saturn's magnetosphere (where ion densities were sufficiently high to make reliable measurements) and argued that the global flow pattern does not seem consistent with the pattern expected for a system dominated by the Dungey cycle; in that, there is a lack of significant inward moving plasma. Rather, an average pattern consisting of strongly azimuthal flows and modest outflow at larger radial distances is observed. A similar pattern was reported by *Kane et al*. [2014] using energetic ion anisotropies.

In this paper we discuss a case study of a field dipolarization observed in Saturn's tail. This event was uncovered during a survey of the Cassini data from 2006, the aim of which was to find evidence of reconnection events, manifested as changes in the north‐south component of the magnetic field. The results of that survey were presented by *Jackman et al*. [2014], with a heavy focus on events tailward of the X line, including 69 plasmoids and 15 tailward moving TCRs. The case presented here is one of the 13 events from that survey which displays a southward turning of the field, indicating a dipolarization of the field while the spacecraft was planetward of the X line. This event is special because in this case all plasma and energetic particle instruments on Cassini were operational and had favorable viewing positions, allowing us to build a multi‐instrument picture of dynamics during this interval. In section 2, we outline the observations; in section 3, we present interpretation and discussion; and in section 4, we summarize.

## 2 Observations

In this paper we use data from the Cassini magnetometer [*Dougherty et al*., 2004], the Cassini Plasma Spectrometer (CAPS) [*Young et al*., 2004], the Magnetospheric Imaging Instrument (MIMI) [*Krimigis et al*., 2004], and the Radio and Plasma Wave Science (RPWS) instrument [*Gurnett et al*., 2004]. The field and particle data sets allow us to build a complete picture of the local magnetosphere, while the radio emission information gives us a global picture of changing magnetospheric dynamics. Specifically, we use two sensors on CAPS. The ion mass spectrometer (IMS) measures energy and composition for ions with energy per charge (*E*/*q*) of 1 eV/e–50 eV/e using an electrostatic analyzer and subsequent time‐of‐flight detector. The electron spectrometer (ELS) has a measurement range of ~0.6 eV/e–28 keV/e, in 63 logarithmically spaced energy levels. Information from two of the MIMI sensors is also used in this analysis: the Charge Energy Mass Spectrometer (CHEMS) and the Ion and Neutral Camera (INCA). CHEMS is able to determine both the mass per charge and the mass of ions, with an energy range from 3 to 236 keV for H^+^ and from 8 to 236 keV for O^+^ ions. Thus, by combining CAPS and MIMI data in this study, we can examine the behavior of ions over an energy range of a few eV up to a few MeV.

**Figure 1:**
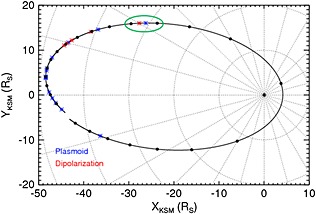
Cassini trajectory for 2006 days 221–245, encompassing one orbit of the spacecraft about the planet as the spacecraft traveled from dawn, through noon, dusk, and then back via midnight. The blue and red asterisks are the timings of plasmoids and dipolarizations, respectively (from the list identified by *Jackman et al*. [2014]). The dipolarization in question for this study occurred on day 232 of 2006, and the green circle surrounds day 232. The black dots are plotted every day from day 222 to day 244.

The event we are focusing on occurred on 2006 day 232 (20 August) when the Cassini spacecraft was ~32 *R_S_* downtail, at a local time of 22:00 and a latitude of ~13.8°. Figure 1 shows one revolution of the Cassini spacecraft about Saturn over a 24 day interval encompassing this event. This figure illustrates the dynamic nature of Saturn's tail region, with the timings of many plasmoids and dipolarizations marked by the blue and red asterisks, respectively. No travelling compression regions were observed during this orbit. These events come from the list of *Jackman et al*. [2014], and they are identified by south‐to‐north and north‐to‐south turnings of the magnetic field, respectively. *Slavin et al*. [2002] used radially aligned spacecraft in Earth's tail to show a close correlation between observation of earthward flow bursts and tailward plasmoid ejections. Furthermore, *Fu et al*. [2012] examined 9 years of data from the Cluster spacecraft at Earth and found the occurrence rate of dipolarization fronts to match that of substorms very closely. If we have an analogous situation at Saturn, we might expect for each observation of a plasmoid that there is an equivalent dipolarization on the other side of the X line and vice versa. Thus, this orbit represents an interval with above average activity, with 14 plasmoids and 5 dipolarizations observed by Cassini in 24 days. Indeed, a plasmoid was observed at 10:01 on day 232, several hours before the event in question here, perhaps indicating an extended interval of tail driving and subsequent relaxation.

### 2.1 Overview of the Interval

**Figure 2:**
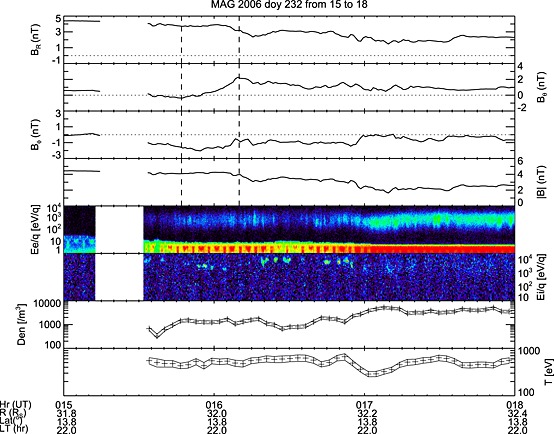
Cassini magnetometer and CAPS data for the interval from 15:00 to 18:00 on 2006 day 232 (20 August). The top four panels show the magnetic field in KRTP coordinates. The vertical dashed lines in the top four panels bracket the main magnetic field change (as identified in the *B_θ_* component) over an ~23 min interval. The next two panels show energy‐time spectrograms from anode 5 of the CAPS electron spectrometer (ELS) and anode 3 of the ion mass spectrometer (IMS) instruments, respectively. The bottom two panels show the electron density and temperature integrated over an actuator cycle (method described in *Arridge et al*. [2009b]). The solid lines above and below the central crosses denote the error bars. ELS measures electrons in the 0.5 eV–28 keV energy range.

Figure 2 shows the magnetic field and plasma data between 15:00 and 18:00 on 2006 day 232. The upper four panels show the magnetic field data at 1 min resolution in the Kronocentric Radial Theta Phi (KRTP) coordinate system. In this spherical polar system, referenced to Saturn's spin axis, the radial component (*B_r_*) is positive outward from Saturn, the theta component (*B_θ_*) is positive southward, and the azimuthal component (*B_φ_*) is positive in the direction of planetary corotation (in a prograde direction). The lower four panels show the time‐energy spectrograms of the electrons and ions and electron densities and temperatures. The effect of spacecraft photoelectrons is visible at the bottom of the electron spectrogram. The electron moments were obtained by building a pitch angle distribution over an ~3 min actuation of CAPS and then numerically integrating the distribution to calculate the number density (*n*) and the parallel and perpendicular temperatures (*T*perp and *T*par). The temperatures reported are total temperatures where *T* = (2*T*perp + *T*par)/3. The error bars indicate the formal errors associated with counting statistics and the background subtraction and are lower limits since they do not include geometric factor uncertainties, or additional systematic effects associated with spacecraft potential corrections [*Arridge et al*., 2009a], pitch angle bin sizes, or the generally incomplete pitch angle coverage (Arridge, personal communication).

At the beginning of the interval shown in Figure 2, the spacecraft was orbiting in a region characterized by a strong, quiet magnetic field and a large positive radial field component. We interpret this region as the northern magnetotail lobe. The average lobe field expected at a radial distance of 32 *R_S_* is 3.92 ± 0.53 nT (3.4 to 4.5 nT) from the fit of *Jackman and Arridge* [2011]. The observed field strength just prior to the data gap was ~4.4 nT at the upper end of this range. A slightly elevated lobe field strength could imply a tail lobe that is loaded with open flux and perhaps primed for reconnection. Data from the Michigan Solar Wind Model (MSwiM) model [*Zieger and Hansen*, 2008; not shown here] indicate that there was a significant increase in solar wind velocity and dynamic pressure at Saturn sometime between day 229 and day 231, although Saturn is far from apparent opposition at this time, and thus, the MSwiM propagations are not very reliable. The ENLIL solar wind propagation model data also indicate a solar wind velocity and dynamic pressure increase at this time (Felici, personal communication), albeit with a time error of up to 4 days. If a solar wind compression hit Saturn's magnetopause, it could stimulate the addition of open flux to the lobes and hence lead to the observed increase in lobe magnetic field strength [e.g., *Jackman et al*., 2004; *Badman et al*., 2005]. The very low fluxes in the CAPS data from 15:00 on day 232 confirm that this is a region largely empty of plasma. There is then a brief data gap (in all instruments) from ~15:15 to 15:35.

Following this data gap, the spacecraft entered a region characterized by fluctuations of the field and a reduction of the radial field component. The CAPS data indicate the presence of denser plasma post data gap, and this may be interpreted as the motion of the spacecraft from lobe field lines before the data gap to plasmasheet‐type field lines immediately after the data gap (suggestive of an expansion or vertical motion of the plasmasheet to higher latitudes up over the spacecraft). The electron temperature in this region is ~400–700 eV (given its time‐dependent behavior), while the density in this region is ~10^3^ el/m^3^.

The *B_θ_* component had been small and positive prior to the data gap, representing “steady state/background” field conditions [*Jackman and Arridge*, 2011]. Following the data gap, it fluctuated close to zero before exhibiting a local minimum of northward (negative) field at ~15:47. This northward field may be understood in terms of regular, wavy plasma sheet motion [*Jackman et al*., 2009a]. Such features are also commonly seen ahead of dipolarization fronts in Earth's magnetosphere [e.g., *Ohtani et al*., 2004]. Following this northward turning, the field sharply and strongly turned southward, with a total field change of ~2.49 nT over 23 min. We interpret this as representative of a dipolarization, where the spacecraft is planetward of the X line. Immediately after the data gap, CAPS observed a narrowly peaked ion distribution (with an energy of 9744 eV), which we show below to be a sunward streaming flow, along with a weak but rather hot (~600 eV) electron distribution that is found to be dominantly bidirectionally field aligned (not shown here). This 600 eV temperature can be compared to the typical quiescent temperature of ~100 eV in the plasma sheet and to the “disturbed” state where the electrons are even hotter (~1 keV) [*Arridge et al*., 2009b]. After ~15:45, i.e., the start of the southward rotation of the field, the electron fluxes increase, and the ion flow is observed at lower energies. Following the end of the dipolarization, the ion flow is seen at higher energies again. The *B_R_* and *B_φ_* components indicate that the field was swept back at this time. From ~16:50 onward, the total field strength and the radial component continued to drop, while *B_ϕ_* was closer to zero, indicating reduced bendback of the field. The character of the electron spectrogram also changed to reflect more “plasmasheet‐like” plasma, and the fast planetward ion flow was no longer present.

### 2.2 Plasma Flow

**Figure 3:**
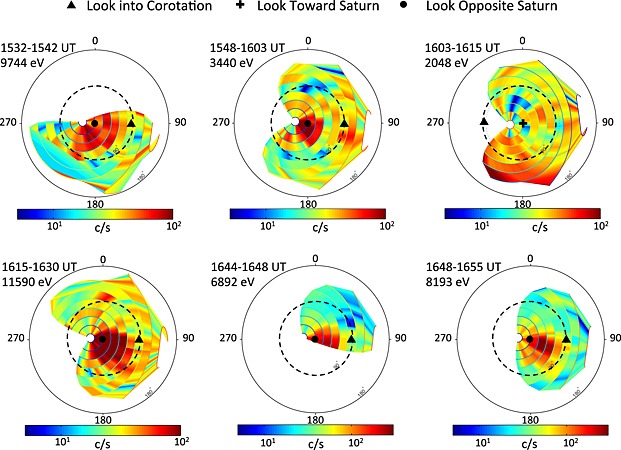
All‐sky images of the ion distribution over various intervals, with times labeled at the top left of each panel. The plots represent slices through the observed distributions at the energy per charge labeled at the top left of each individual plot (the distribution peak). With the exception of the third panel, for 16:03–16:15 (reversed for display purposes), the look direction away from Saturn is in the center (i.e., for inward moving flow). The radial distance from the center is proportional to the polar angle of the viewing direction relative to the center direction (away from Saturn for all panels except the third). Thus, the entire outer circle corresponds to the look direction away from Saturn (toward Saturn for panel 3, 16:03–16:15), and the dashed circle halfway to the outer boundary indicates look directions that are 90° away from Saturn's direction. The azimuth in the plots (indicated by the angle markings around the circumference of the outer circle, given in degrees) corresponds to the azimuth of the look direction relative to a meridian containing the direction to Saturn and Saturn's spin (and magnetic dipole) axis, measured about the axis pointing toward Saturn. The corotation direction (on the right‐hand side of each plot, other than the 16:03–16:15 panel, where it lies on the left‐hand side) is marked by a filled triangle. Planetward flows are seen between the corotation direction and the anti‐Saturnward look direction (i.e., inside of the dotted 90° circle or outside that circle for 16:03–16:15). The hydrogen speeds for each interval (with increasing time) are 1.37 × 10^3^, 812, 627, 1.49 × 10^3^, 1.15 × 10^3^, and 1.25 × 10^3^ km s^−1^.

The data presented in Figure 2 indicate that a dynamic event occurred in the magnetotail, the consequences of which are observed by the spacecraft after the data gap. As mentioned above, the narrow (in energy) ion population seen in CAPS is found to be streaming in the Saturnward direction. This streaming can be seen in the panels of Figure 3, which show the color‐coded ion counts within the field of view of the CAPS instrument for several time intervals between ~15:30 and 16:55. Each panel is drawn for the energy of the peak counts during that interval. From this figure, we can see that the spacecraft was rolling during this interval, and thus, the CAPS instrument had good all‐sky coverage. Anodes 1 and 2 were looking outward (downtail), and anodes 7 and 8 were looking inward (planetward). From the CAPS IMS data, we can infer that even though the energy of the peak in each time interval varied (as shown in Figure 3), the flows were directed consistently planetward with a slight component in the corotation direction and with a velocity of ~600–1500 km s^−1^ (see also *McAndrews et al*. [2009a] for preliminary analysis of this interval). These flow speeds are inferred from the energy of the peak counts (highest‐energy flows in the 15:32–15:42 and 16:15–16:30 intervals) and thus represent an upper limit to the flow speed (because there may be some contribution to the energy from the thermal speed; however, as the flows are relatively narrow in most cases, the thermal speed is small compared to the flow speed). These flow speeds are consistent with both the MIMI INCA and CHEMS data. *McAndrews et al*. [2009b, 2014] presented a very comprehensive survey of plasma ions at Saturn and found that ion flows are typically 150–200 km s^−1^ and predominantly in the corotational direction out to 30 *R_S_*, with some outward flow beyond this distance. Thus, the flows observed here represent a significant deviation from the usual behavior in the tail plasma sheet. When seen at Earth, such fast, planetward flowing populations at the edge of the plasma sheet can be associated with reconnection occurring tailward of the spacecraft (or occasionally can be explained as reflection of particles from a dipolarization front [e.g., *Zhou et al*., 2012]). In this case, based on our multi‐instrument analysis, we adopt the former interpretation that reconnection ongoing tailward of the spacecraft is producing the observed directional flows.

### 2.3 Plasma and Energetic Particle Composition

Plasma composition can be a key diagnostic of the source of the field‐aligned flows. Specifically, if we interpret the directional flows as originating in tail reconnection, the presence or absence of water group ions should help distinguish between reconnection of open lobe field lines (Dungey reconnection) and internal reconnection of closed field lines (Vasyliunas reconnection). Planetward of the X line, if reconnection of open field lines occurred, we may expect a flow of light ions (e.g., H^+^ or He^++^ from the solar wind, or other light ions originally of ionospheric origin, cf., the polar wind model of *Glocer et al*. [2007]). If, on the other hand, reconnection occurred on closed field lines holding magnetospheric plasma, we would expect the outflow from the reconnection site to contain water group ions. Water group ions exclusively originate from within the magnetosphere, and as such, they are an excellent tracer of reconnection on closed field lines carrying internally generated plasma. We might expect the heavy ions to be somewhat depleted relative to light ions on the planetward side of the X line due to equatorial confinement. However, the larger‐scale height of the higher‐energy ions means there should still be enough of the heavy water group ions to serve as tracers of internal reconnection.

**Figure 4:**
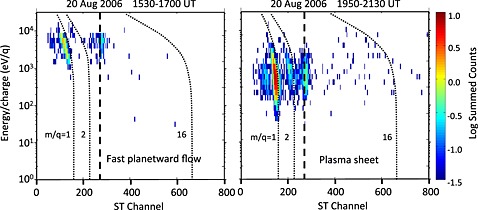
Energy per charge versus time‐of‐flight spectrograms from the CAPS/IMS spectrometer. The left‐hand panel is for the interval from 15:30 to 17:00 and the right‐hand panel for 19:50–21:30. The straight line indicates the locus of artificial counts from secondary electrons produced when ions strike the CAPS/ELS detector (see *Young et al*. [2004]).

For information regarding the plasma composition in the directional planetward flows, we refer to Figure 4, which shows two energy per charge versus time‐of‐flight spectrograms from CAPS/IMS: the first for the interval from 15:30 to 17:00, which contained the flows, and the second for a “typical” plasma sheet interval several hours after the event (19:50–21:30). By way of context, the right‐hand panel shows the species typically present in the plasma sheet: a combination of light (H^+′^ and *m*/*q* = 2, which could be H_2_^+^ or He^++^) and water group (O^+^, OH^+^, H_2_O^+^, and H_3_O^+^) ions. The populations within the planetward directional flows are in stark contrast to this. In their case (left‐hand panel from 15:30 to 17:00), CAPS detects light ions, primarily H^+^ (*m*/*q* = 1) and a few with *m*/*q* = 2 (He^++^ or H_2_^+^). The energy of the hydrogen ions is ~3.4–11.6 keV (see also Figure 3). There is a distinct absence of heavier water group (W^+^) ions at this time (some arrive at the spacecraft after ~17:00 as the more isotropic plasma sheet returns, but they are absent throughout the entire dipolarization/energization interval). There are two possible reasons for the lack of observation of heavy ions. First, it is possible that the heavy ions are not visible if they are centrifugally confined closer to the equator and thus do not reach the latitude of the spacecraft (13.8°) in this case. However, we note that the magnetic field and plasma data indicate that the plasmasheet expanded over the spacecraft and thus the spacecraft found itself sampling a heated outer plasmasheet population, which should contain some oxygen. A second reason for the lack of W^+^ observation is that if oxygen ions are present and travel at the same speed as the hydrogen ions in this example, their energy would be ~54–186 keV, above the range visible to CAPS (maximum of 28 keV).

**Figure 5:**
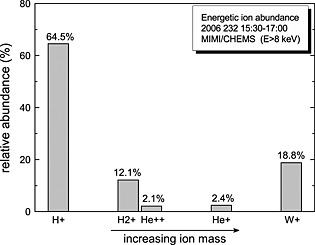
Relative abundance of energetic ions (mass‐per‐charge data, 8–235 keV/e) obtained with MIMI/CHEMS between 15:30 and 17:00 of day of year 232/2006. The number of counts for each ion species is normalized to the total. The W^+^ ion group consists of O^+^, OH^+^, H_2_O^+^, and H_3_O^+^. Ion species are presented in increasing mass order. Although they have the same mass per charge (*m*/*q* = 2), H_2_^+^ and He^++^ can be separated due to their different mass (2 amu and 4 amu, respectively).

In order to explore the composition of higher‐energy particle populations, we first examine data from MIMI CHEMS, which measures ions just above the CAPS range, between 3–236 keV per charge for H^+^ and 8–236 keV for O^+^. Figure 5 displays a histogram showing relative energetic ion abundances from the CHEMS instrument. The CHEMS data allow us to distinguish the *m*/*q* = 2 species from the CAPS time‐of‐flight plot above, and we find that H_2_^+^ (magnetospheric constituent) is ~6 times more abundant than He^++^ (solar wind constituent) when averaged over the interval from 15:30 to 17:30, indicating a magnetospheric composition of the energetic ion population. The energy of the water group (containing oxygen) is precisely in the range predicted above (~54–186 keV).

**Figure 6:**
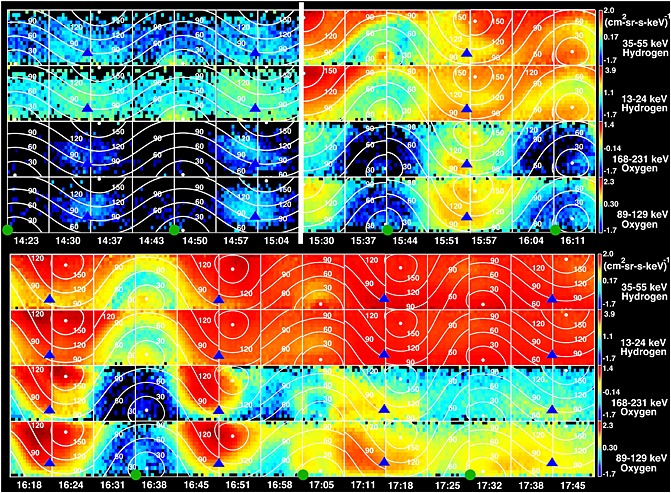
Angular distributions for hydrogen and oxygen measured by the INCA sensor for the time period of 14:23–17:45. The time period is broken into two panels, where the top two rows in each panel show hydrogen, while the bottom two rows show oxygen. As Cassini rolls about its *Z* axis during this observation, a full 360°×120° angular distribution is obtained every four INCA images (about 27 min). Each INCA image of 16 × 16 pixels consists of the color‐coded H^+^ or O^+^ intensities (c/cm^2^ sr s keV) and the 30°, 60°, 90°, 120°, and 150° pitch angle isocontours denoted by the thin white lines. Note that the logarithmic color bar has a different scale for each species and energy. The thick vertical white line in the top denotes the data gap after 15:04. The blue triangles indicate the direction from which one would expect enhancements in the intensities produced by flow in the nominal corotation direction. The anisotropy is consistent with a flow direction fairly close to corotational prior to 15:04 UT and after 17:00 UT. The green circles are shown in the approximate location of Saturn in the INCA field of view for the bottom row of angular distributions in each panel. Flow away from Saturn would appear near the green circles (which are also close to field aligned), whereas flows toward Saturn would appear near the top of each frame.

In order to explore the energetic particles in more detail, Figure 6 shows data from INCA for hydrogen (top two panels of each row, 35–55 keV and 13–24 keV) and oxygen (bottom two panels of each row, 168–231 keV and 89–129 keV) species separately, over time intervals spanning from 14:23 to 17:45. CHEMS and INCA agree well throughout this interval. The data from 14:23 to 15:04 confirm the quiet, empty lobe‐like picture presented above from magnetometer (MAG) and CAPS. Interestingly, there is a weak O^+^ and a weak H^+^ population seen in two of the lobe spectra. These may be due to finite gyroradius remote sensing of the plasmasheet because they appear to be nongyrotropic, fill less than 180° in gyrophase, and are peaked at 90° pitch angles. Immediately following the data gap, there is evidence of an anisotropic flow toward the planet. This flow is initially present in both H^+^ and O^+^, with an increase in the strength of the O^+^ ions after ~15:51 likely associated with a further increase in the flow velocity (note the accompanying increase in the CAPS directional flow energy in Figure 2). A similar delay is seen by CHEMS, but we note that these water group ions appear as soon as CHEMS is in a favorable configuration to see them. While the anisotropy of the energetic ions is indeed strong, it has a relatively broad pitch angle distribution (~90–180°) and thus might be termed a “flow burst.” Even within the MIMI observations, we see differences in the anisotropy width between species. The anisotropy is slightly more confined in oxygen than in hydrogen, because the bulk flow energy of the oxygen is higher and thus represents a larger fraction of the oxygen total (bulk flow + thermal) energy (yielding a more dominant flow anisotropy). The anisotropic flow in both hydrogen and oxygen continues through to 16:45. Later in the day, from 17:11, there is evidence of some tailward flow, with an increasingly isotropic distribution thereafter and a return to a lower intensity plasma sheet.

### 2.4 Global Magnetospheric Dynamics

**Figure 7:**
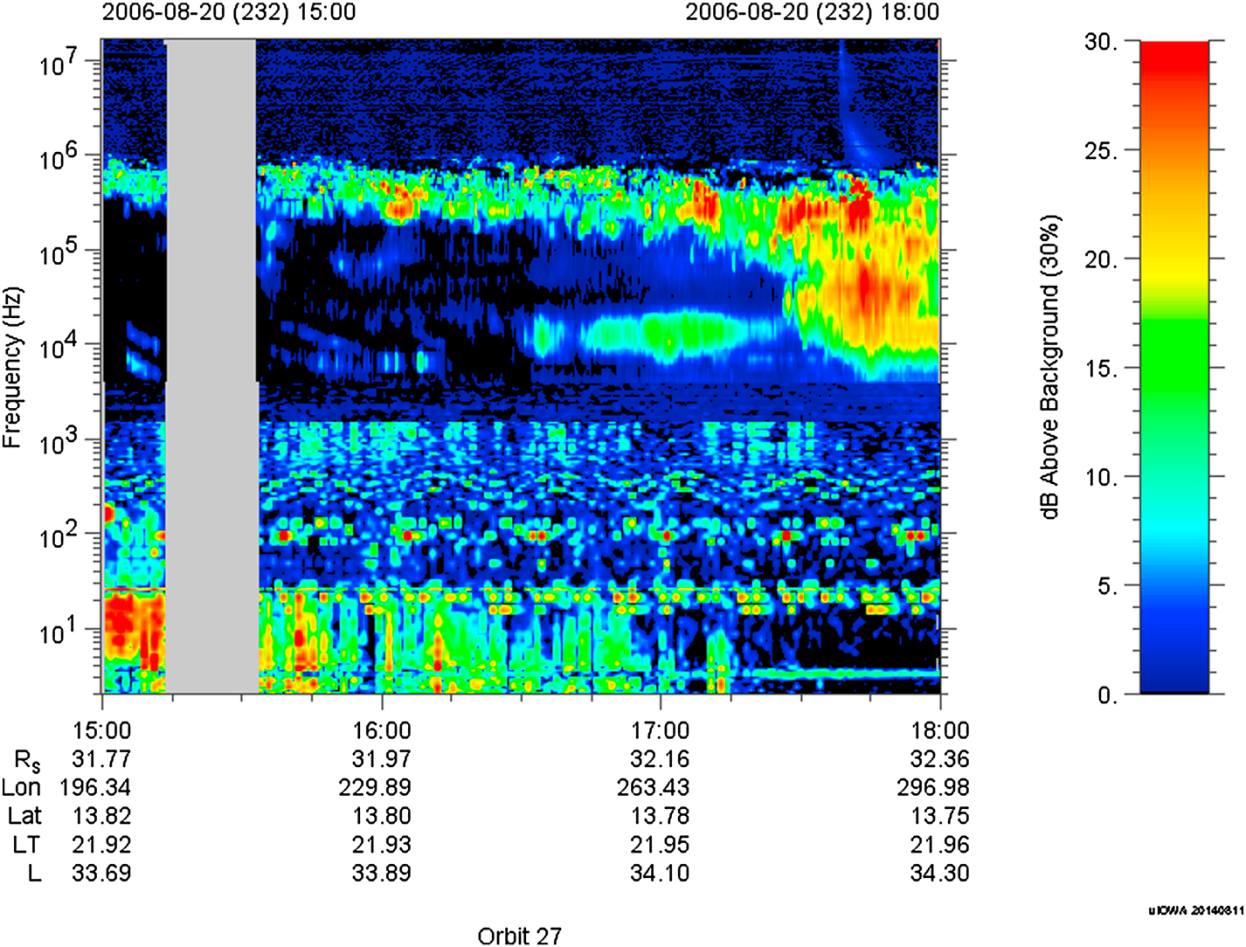
Frequency‐time spectrogram from the RPWS instrument for the interval from 15:00 to 18:00 on day 232 of 2006.

In order to explore the effect that this reconnection event may have had on global magnetospheric dynamics, in Figure 7, we show data from the Radio and Plasma Wave Science (RPWS) instrument in the form of a frequency‐time spectrogram from 15:00 to 18:00. The dipolarization as identified from the magnetometer data began at ~15:47, continuing until ~16:10, with further directional ion flows and disturbed field for some time thereafter, perhaps implying ongoing reconnection. From Figure 7, we see that there is some emission at lowest frequencies of 10–30 kHz from ~16:30, which can indicate emission from a high‐altitude source [e.g., *Lamy et al*., 2013]. This is followed by a strong intensification and low‐frequency extension of the SKR emission from ~17:20 onward. We note that the timing of this SKR emission observation may have been delayed due to observational issues whereby the spacecraft needs to move into the appropriate viewing region in order to be within the SKR emission cone [*Lamy et al*., 2008]. SKR onset at the planet may thus occur earlier than observation at the spacecraft. Furthermore, as the SKR is a current‐driven instability, there is a finite time (minimum of the Alfvén transit time) from the onset of reconnection in the tail for the current coupling with the ionosphere to be established and for sufficient current to build up in the ionosphere to drive the SKR. Alfvén speeds in the lobes can be >4000 km/s [*Arridge et al*., 2009b], but taking a conservative estimate of 1000 km/s, the one‐way Alfvén travel time to traverse 30 *R_S_* is ~30 min. For these reasons, we can understand a delay between in situ observations of reconnection in the tail and the appearance of likely associated SKR emission. *Mitchell et al*. [2005] noted that bursts of ENA activity were well correlated with enhancements in the intensity of the SKR, while *Jackman et al*. [2009b] focused in particular on the generation of a potential drop at higher altitudes that would lead to the observed low‐frequency extension of the radio emission. The ~90 min time delay between the observation of the start of the dipolarization and the response of the radio emissions is within the correlation time scales explored by authors such as *Mitchell et al*. [2005] (but as noted above, this delay may be shorter than observed). What does this imply for the speed of the dipolarization front? Given that the SKR is observed to brighten from ~17:20, we can thus estimate the speed of the dipolarization front (southward turning starting at 15:47) if we assume transport from the site of the dipolarization (32 *R_S_*) in to ~15 *R_S_*, where the ENA brightenings that are typically associated with SKR intensifications occur. *Mitchell et al*. [2009] noted that at this distance, pressure gradients associated with the injected plasma drive strong enough field‐aligned currents to generate SKR. A population initiated at 32 *R_S_* at 15:47 would have to travel at 183 km s^−1^ to reach 15 *R_S_* by 17:20. We note that this bulk flow velocity is much slower than the directional flow speeds but is of the appropriate order of magnitude when compared to the speed of terrestrial dipolarization fronts [e.g., *Nakamura et al*., 2002; *Runov et al*., 2009].

## 3 Discussion

In this paper we have presented magnetic field, low‐energy plasma, and energetic particle observations, as well as radio and plasma wave data for a dipolarization event observed by the Cassini spacecraft in Saturn's tail. Below, we discuss the observations in terms of a timeline of the event.

### 3.1 Timeline of the Event

**Figure 8:**
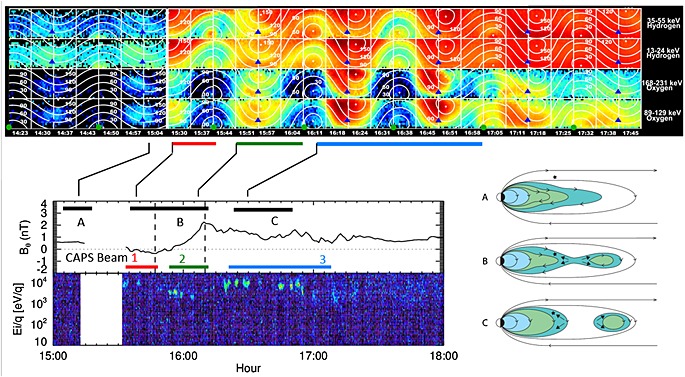
MIMI INCA, magnetometer, and CAPS data combined with a schematic figure to aid interpretation of the interval. (top) The angular distributions for hydrogen and oxygen obtained by the INCA sensor for the time period of 14:23–17:45, in a similar format to Figure 6, with hydrogen in the top two rows and oxygen in the bottom two rows (although shown as one continuous stretch of data in this case). (bottom left) The north‐south (*B_θ_*) magnetic field component and the CAPS IMS spectrogram in the same format as Figure 2 from 15:00 to 18:00. The horizontal black bars marked A, B, and C in the magnetometer data panel (matched to the corresponding MIMI data above) show the intervals that for which the magnetotail topology is sketched schematically in the top right of the figure. The schematic sketches on the bottom right are meridional cuts through the nightside magnetosphere. The star in each panel represents the spacecraft.

Figure 8 includes a combination of MIMI‐INCA, CAPS, and magnetometer data, along with a schematic illustration of three intervals (A, B, and C) which correspond to distinct stages of the reconfiguration of the magnetotail in this event. We discuss the timeline of observations with reference to Figure 8 below.

In interval A from Figure 8, representing the interval around 15:04 prior to the data gap, the quiet magnetometer data, “empty” CAPS spectrograms, and low fluxes in MIMI indicate that Cassini was in the lobes. Any observation of O^+^ or H^+^ ions by INCA during this interval can be attributed to finite gyroradius “leakage,” whereby energetic ions can escape from closed plasmasheet field to adjacent lobe field lines. We note, for example, that the gyroradius of a 100 keV O^+^ ion in a field strength of 5 nT is 0.6 *R_S_*, and thus, it can be easy for such energetic particles to cross from the higher‐latitude plasmasheet to the nearby lobe.

Interval B from Figure 8 corresponds to the interval from 15:30 to 16:10 after the data gap and encompasses the northward (15:30–15:44) to southward (15:44–16:10) turning of the field, which we interpret as a dipolarization associated with reconnection tailward of the spacecraft. This magnetic signature is accompanied by some intriguing low‐energy plasma and energetic particle flows.

Immediately after the data gap, the field strength decreased while field fluctuation increased. This combined with the appearance of sunward ion fluxes and few hundred eV electrons indicates that the spacecraft had entered a closed field line region. Several authors have noted that there is not always a smooth transition between the lobes and the plasma sheet [*Arridge et al*., 2009b; *Jackman and Arridge*, 2011; *Simon et al*., 2010; *Sergis et al*., 2011], with different particle populations and different energies having different scale heights and behaviors. The electron population observed immediately after the data gap in this case is consistent with what *Arridge et al*. [2009b] have called the “outer plasma sheet.” The temperature immediately after the data gap was ~500 eV, with electron densities of a few hundred m^−3^. The sunward directional ion flows observed during this interval (speeds of ~1300 km/s between 15:32 and 15:42) are consistent with flow from a reconnection site tailward of the spacecraft.

The addition of the MIMI data provides further information on the behavior of plasma and energetic particles at this time. In addition to the sunward directional ion flow seen clearly in CAPS, MIMI sees sunward flow and an additional tailward streaming hydrogen component around 15:37, particularly at the highest energies. A similar population was present (but weaker) before the data gap, though at somewhat lower energies. The planetward flowing plasma between 90 and 180° pitch angle has a much softer spectrum than the simultaneously observed tailward flow. The energetic oxygen (in the bottom of the MIMI data in Figure 8) shows only planetward flow. The planetward flow of energetic hydrogen and oxygen observed in this case is consistent with the spacecraft position planetward of a downtail reconnection site. Direct connection of the spacecraft to an active reconnection site cannot be uniquely established on the basis of the data presented here; however, the observations are certainly consistent with the products of reconnection.

With regard to the tailward flow of energetic hydrogen, a simple analogy with Earth might interpret this as bidirectional streaming indicative of a PSBL‐type structure [e.g., *Eastman et al*., 1984; *Walsh et al*., 2011] as particles which mirror closer to the planet are reflected back downtail. However, the strongly field aligned nature of the tailward H^+^ at this time is inconsistent with a population that has mirrored close to the planet, as is its presence on lobe field lines prior to the data gap. Rather, we interpret it as a population of ionospheric ions which have been accelerated at low altitude in a high field region. The tailward population (highly field aligned, hydrogen only) is rather different to the planetward population (not well ordered by the field, hydrogen, and oxygen). Ionospheric outflows are generally composed of light ions [e.g., *Glocer et al*., 2007; *Nagy et al*., 2009], mainly because the scale heights of light ions are higher than they are for heavy ions, placing the light ions at sufficiently high altitudes to be accelerated by waves and/or field‐aligned electric fields. Such a population is likely produced in a “pressure cooker” region driven by intense field‐aligned currents associated with the event [e.g., *Carlson et al*., 1998; *Mitchell et al*., 2009].

Interval C of Figure 8 corresponds to the interval from 16:10 to 17:00 when the initial magnetic dipolarization signature had finished. CAPS continued to observe further planetward ion flows, indicating that Cassini continues to be immersed in flows from ongoing reconnection. Indeed, the directional CAPS flows observed at this time are some of the fastest of the interval, with speeds up to 1500 km/s (15:15 to 16:30) and composition primarily light ions (W^+^ ions are not observed until 17:00 at the earliest). The INCA data show a substantial population of W^+^ at energies above the CAPS energy range and are consistent with this picture of high flow speeds. Meanwhile, the magnetic field remains disturbed. The CAPS directional flow speeds are much faster than typical plasma flows in this region of the tail and much faster than the ~200 km/s return flow observed in the case study by *Masters et al*. [2011].

Finally, from 16:58 onward, the energetic particle data show an increasingly isotropic distribution, which, combined with the CAPS observations, indicate a return to more typical plasma sheet. Thus, the in situ spacecraft data from this local time meridian indicate that the magnetotail is returning to a more quiescent state as in situ reconnection signatures cease. However, the RPWS instrument registers strong Saturn kilometric radiation emission and low‐frequency extension from ~17:20, the likely response of the auroral zone to currents driven by the injection from the tail, and perhaps indicating ongoing activity elsewhere in the nightside magnetosphere.

### 3.2 Interpretation of the Event

#### 3.2.1 Long‐Duration/Quasi‐Steady Reconnection

The distinguishing feature of this event in terms of the magnetic field observations is the 23 min dipolarization signature, indicative of field lines contracting back toward the planet following reconnection. However, the plasma and energetic particles also provide an intriguing insight into the magnetospheric dynamics during this interval. Several strong planetward energy‐collimated flows are seen in CAPS, with similar flows in MIMI. The fact that they are persistently sunward (with the exception of a small interval of tailward energetic H^+^ that may be associated with the tail reconnection but only through field‐aligned currents coupling with the ionosphere) indicates that we are seeing the response to reconnection that is occurring tailward of the spacecraft. Moreover, the presence of substantial fluxes of energetic O^+^, especially after ~16:20, indicates that the reconnection involves internally loaded magnetospheric field lines, i.e., Vasyliunas‐type reconnection. The dominance of H_2_^+^ over He^++^ as seen from the CHEMS data supports this picture. The directional flows are relatively long lived, with energized ions persisting for ~1.5 h, much longer than the ~23 min dipolarization field signature. The long duration of the planetward flow interval represents ~14% of a planetary rotation, and a corotational flow of 200 km/s could move up to ~2.1 h in local time during this period, covering a significant fraction of the nightside of the planet in this time. If the duration over which the CAPS directional flows are observed is taken to represent the extent of tail activity, this could thus imply quasi‐steady reconnection in Saturn's tail for a significant fraction of a planetary rotation, which could have a significant impact on the topology of the field and the nature of plasma flows in the region.

Such long‐lasting reconnection has been reported before, where *Thomsen et al*. [2013] observed a long‐lasting inflow event, with water‐depleted plasma observed moving planetward over ~2.5 h. They suggested that this event might represent the dipolarization exhaust from a quasi‐steady reconnection event tailward of the spacecraft, with the exhaust continuing as new longitudinal sectors rotated into the reconnection region. Such long‐lived reconnection could be part of the answer to the mass budget imbalance discussed by *Jackman et al*. [2014], whereby large‐scale plasmoid break‐off has been shown to be insufficient to match the mass added to the magnetosphere. The mean duration of a plasmoid event from that study was ~17.71 min, while plasmoids observed within a “viewing region” of the nightside of the planet are estimated to remove ~2.59 kg/s of plasma, a small fraction of the estimated ~100 kg/s loaded into the magnetosphere by Enceladus [*Bagenal and Delamere*, 2011]. If reconnection is ongoing for an hour or more, a significant amount of mass could be released in this time. At the end of *Thomsen et al*.'s [2013] dipolarization event, the character of the plasma changed and the spacecraft observed a return to higher densities, larger W^+^ fraction, and more typical azimuthal flow. They interpreted this as signifying the arrival of a new longitudinal sector at the spacecraft where reconnection was not occurring. We see a similar pattern in our event (after interval C from Figure 8) where the tail returns to a more quiescent state, with isotropic plasma distributions and more typical plasma sheet populations with less disturbed field.

#### 3.2.2 Planetward Flow: A Relatively Rare Observation at Saturn

In this paper we present an example of very strong planetward flow. How unusual is this event? *McAndrews et al*. [2009b, 2014] presented a bulk flow map of Saturn's nightside over radial distances from 10 to 50 *R_S_*. Their results showed that flow was primarily in the corotation direction. They found no evidence for significant inflowing plasma (despite favorable instrument pointing on many occasions), although they noted that they expect planetward flow to exist at least occasionally in order to account for the low‐density plasma populations that they observe on Saturn's nightside. *Thomsen et al*. [2013, 2014] analyzed Cassini's dusk and nightside orbits for intervals where the CAPS instrument viewing was balanced inward versus outward and found “numerous” significant examples of planetward plasma flow, primarily premidnight and inside of 25 *R_S_*, with one such event identified as a dipolarization. However, flow speeds were generally lower than corotation. Thus, the suggestion from those studies was that tail reconnection in the dusk sector has not yet proceeded to involve lobe field lines. It is also likely that the outward stretching mass‐loaded flux tubes in Saturn's middle magnetosphere may inhibit the Saturnward return flow of newly reconnected flux in all but a few cases. Most recently, *Kane et al*. [2014] surveyed the energetic ion anisotropies at Saturn and found some evidence of inflow at local times greater than 3 h (the interval not covered in *Thomsen et al*.'s [2013] study). These studies are illustrations of the additional phenomenology present in the Saturn system compared to Earth. The production of large amounts of plasma within the magnetosphere and the rapid rotation of the planet itself lead to the strong corotational influence, with flow maps indicating predominantly azimuthal motion (as opposed to radially inward or outward plasma flow following large‐scale reconnection events). We thus infer that the case presented here is a rather rare observation: an example of strong, narrow, fast planetward flow, indicative of the occurrence of reconnection downtail of the spacecraft. We note, however, that the associated SKR signature is a relatively common one, and thus, it is possible that this type of event occurs more commonly than we can observe in situ. Perhaps the paucity of such in situ observations is linked to the small amount of time that Cassini spends near the plasma sheet‐lobe boundary.

## 4 Summary and Conclusions

In this paper we have presented a rare observation of strong planetward flow following a reconnection episode in Saturn's tail. The reconnection event involves a dipolarization of the field, the result of reconnection at a site tailward of the spacecraft. The dipolarization precedes by slightly more than an hour the occurrence of an SKR enhancement like those that have been previously associated with plasmoid formation and release. Prior to the appearance of the dipolarization front at the spacecraft, the effect of this reconnection is sensed through the appearance of strong planetward flows of H^+^ ions (with speeds of 600–1500 km/s), as well as planetward flow of energetic hydrogen and oxygen. In addition, beams of field‐aligned hydrogen ions flowing tailward were observed near the lobe boundary, presumably due to ionospheric outflow related to reconnection‐driven currents. The reconnection episode as inferred from the planetward directional flow duration lasts on the order of ~1.5 h, a significant fraction of a planetary rotation. The continuing presence of energetic O^+^ ions throughout the event demonstrates that this must be a case of long‐lasting Vasyliunas‐type reconnection occurring beyond 32 *R_S_* in the premidnight region, perhaps indicating quasi‐steady reconnection of the type suggested by *Thomsen et al*. [2013]. Because of the persistent presence of O^+^, we find little evidence for lobe involvement in the reconnection.
